# Trans-Synaptic Spread of Tau Pathology *In Vivo*

**DOI:** 10.1371/journal.pone.0031302

**Published:** 2012-02-01

**Authors:** Li Liu, Valerie Drouet, Jessica W. Wu, Menno P. Witter, Scott A. Small, Catherine Clelland, Karen Duff

**Affiliations:** 1 Department of Pathology and Cell Biology, Taub Institute for Alzheimer's Disease Research, Columbia University, New York, New York, United States of America; 2 Kavli Institute for Systems Neuroscience and Centre for the Biology of Memory, Norwegian University of Science and Technology, Trondheim, Norway; 3 Department of Neurology, Taub Institute for Alzheimer's Disease Research, Columbia University, New York, New York, United States of America; 4 Department of Psychiatry, New York State Psychiatric Institute, New York, New York, United States of America; Boston University School of Medicine, United States of America

## Abstract

Tauopathy in the brain of patients with Alzheimer's disease starts in the entorhinal cortex (EC) and spreads anatomically in a defined pattern. To test whether pathology initiating in the EC spreads through the brain along synaptically connected circuits, we have generated a transgenic mouse model that differentially expresses pathological human tau in the EC and we have examined the distribution of tau pathology at different timepoints. In relatively young mice (10–11 months old), human tau was present in some cell bodies, but it was mostly observed in axons within the superficial layers of the medial and lateral EC, and at the terminal zones of the perforant pathway. In old mice (>22 months old), intense human tau immunoreactivity was readily detected not only in neurons in the superficial layers of the EC, but also in the subiculum, a substantial number of hippocampal pyramidal neurons especially in CA1, and in dentate gyrus granule cells. Scattered immunoreactive neurons were also seen in the deeper layers of the EC and in perirhinal and secondary somatosensory cortex. Immunoreactivity with the conformation-specific tau antibody MC1 correlated with the accumulation of argyrophilic material seen in old, but not young mice. In old mice, axonal human tau immunoreactivity, especially at the endzones of the perforant pathway, was greatly reduced. Relocalization of tau from axons to somatodendritic compartments and propagation of tauopathy to regions outside of the EC correlated with mature tangle formation in neurons in the EC as revealed by thioflavin-S staining. Our data demonstrate propagation of pathology from the EC and support a trans-synaptic mechanism of spread along anatomically connected networks, between connected and vulnerable neurons. In general, the mouse recapitulates the tauopathy that defines the early stages of AD and provides a model for testing mechanisms and functional outcomes associated with disease progression.

## Introduction

**Figure 1:**
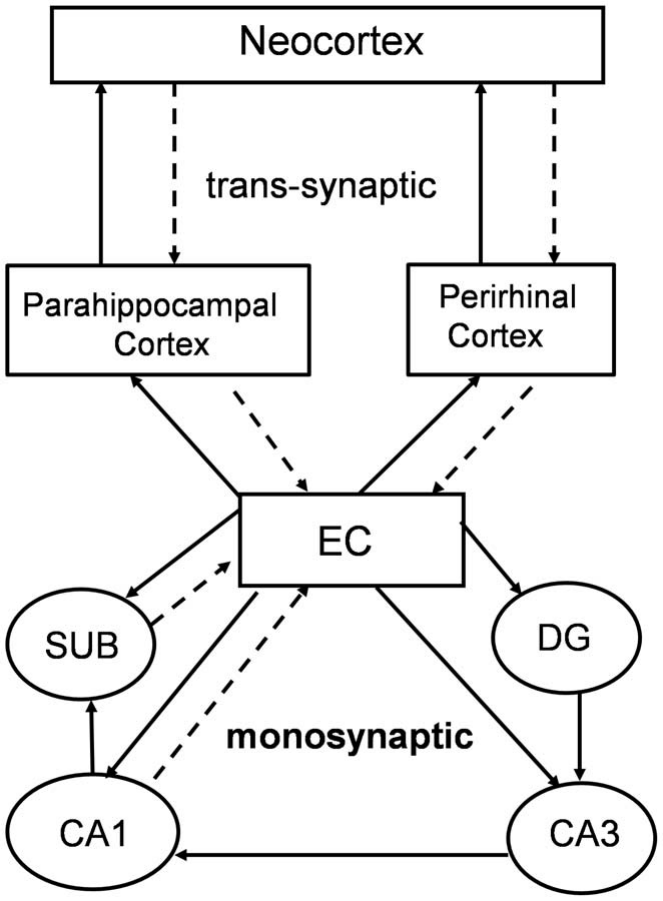
Monosynaptic and trans-synaptic cortico-hippocampal and cortico-cortico connections. Solid lines indicate projections radiating out from the EC, dotted lines indicate projections to the EC. Monosynaptically connected regions are connected across one synapse. Trans-synaptic regions are separated by more than one synapse.

AD is characterized neuropathologically by the presence of amyloid-β containing plaques and neurofibrillary tangles (NFTs) composed of aggregated, fibrillar, hyperphosphorylated forms of the microtubule-associated protein tau. The earliest stages of the disease show accumulation of abnormal tau in the entorhinal cortex (EC) whereas later stages show accumulation in the hippocampus followed by neocortical areas [1]. As shown in Fig. 1, the EC is monosynaptically connected to other hippocampal subregions and it is trans-synaptically connected with affected regions in the temporal and parietal lobes [2], [3]. One of the most intriguing and poorly explored questions in the field is whether pathology, and/or dysfunction of the EC initiates anatomical progression of the disease, or whether pathology and/or dysfunction in extrahippocampal areas develops independently, and is unrelated to events occurring in the EC. There are a number of interesting, albeit circumstantial observations that support the trans-synaptic spread hypothesis for AD both in terms of pathology development and functional outcome. First, by simply charting the anatomical distribution of the pathology in human post-mortem tissue, the affected areas appear to be trans-synaptically linked [4]. Second, functional imaging studies in non-human primates have shown that lesioning the rhinal cortex (perirhinal and entorhinal) causes secondary dysfunction in the temporal and parietal lobes [5]. Currently available AD transgenic mouse models do not allow for studies of disease circuitry and progression as they generally overexpress APP or tau in inappropriate areas, or at high levels throughout the brain making it hard to identify temporal and spatial progression between vulnerable areas. To address this shortcoming, we have generated a transgenic mouse model with restricted expression of pathological human tau that predominates in the entorhinal cortex. We have performed a detailed histopathological analysis of the mice to map the change in distribution of tauopathy as the mice age. Our data support a temporal and spatially defined mechanism of trans-synaptic spread along anatomically connected networks, between connected and vulnerable neurons that replicates the early stages of AD.

## Results and Discussion

### Histopathology studies demonstrate progressive tauopathy radiating from the EC

**Figure 2:**
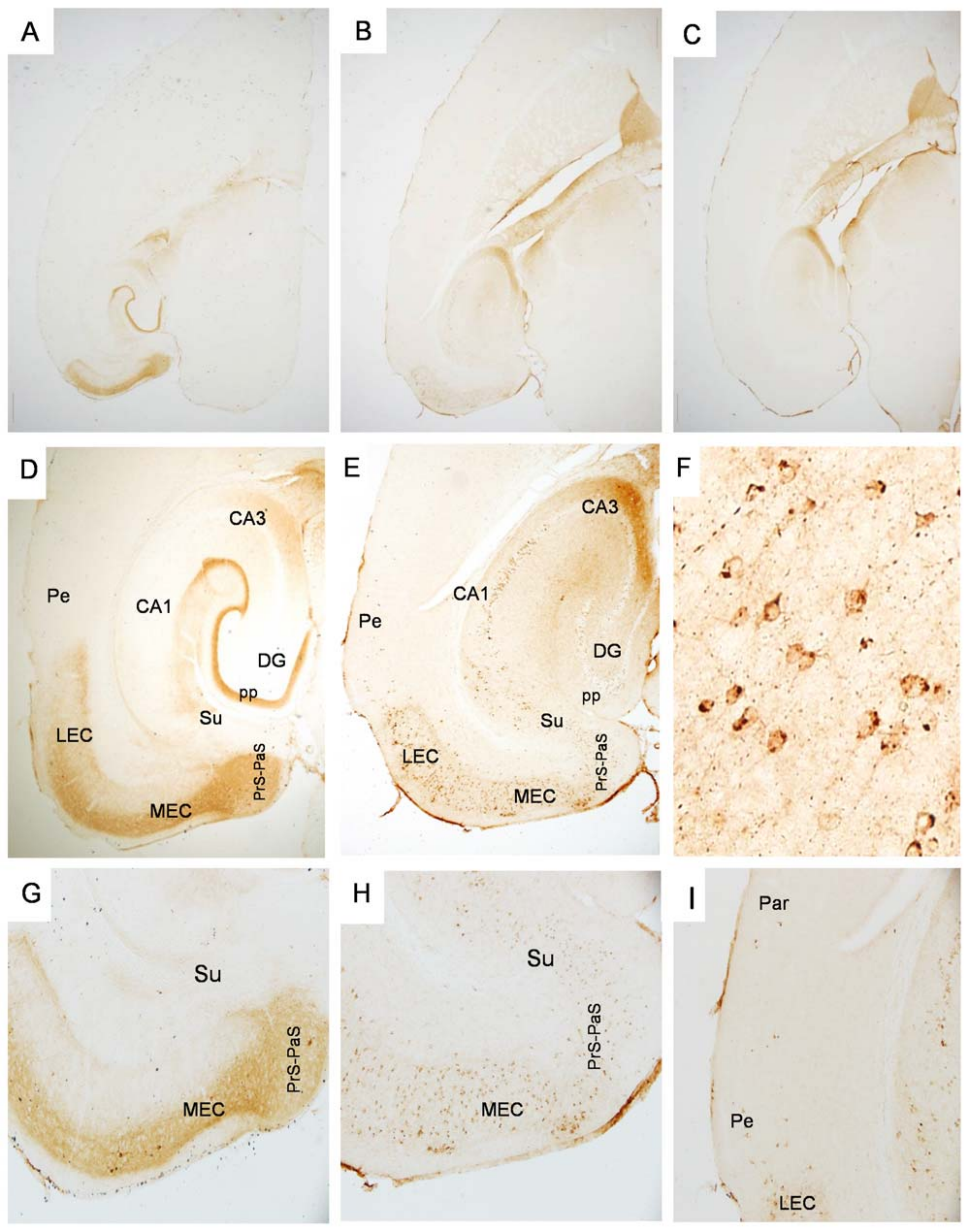
Progressive spread of tauopathy in NT mice identified by antibody MC1. Fig. 2A shows tau immunolabeled with the human tau specific, conformational antibody MC1 in a young NT mouse at low power, and higher power (Figs. 2D, G). Fig. 2B shows MC1 immunolabeling in an old NT mouse at low power, and higher power (Figs. 2E, H). Fig. 2F shows high power image of cells immunolabeled with MC1 within the MEC. Old NT mice show extensive accumulation of human tau in cell bodies in the EC and subiculum (Fig. 2H), and in synaptically connected areas in the hippocampus and neocortex (Fig. 2E). Fig. 2I shows accumulation of human tau in neurons of the perirhinal cortex and into the parietal region in the old NT mouse. Note the lack of neurite staining in the perirhinal cortex compared to the LEC. Fig. 2C shows lack of immunolabeling with the human specific antibody in an old, littermate control mouse (single transgenic tau responder mouse, no tTA) except for the non-specific staining of the fornix that was seen with all antibodies. MEC = medial entorinal cortex, LEC = lateral entorhinal cortex, Pe = perirhinal cortex, Par = parietal cortex, DG = dentate gyrus, CA1, CA3 = CA fields of hippocampus, Su = subiculum, Prp-PaS = pre-parasubiculum, pp = perforant pathway endzone. Figs. 2A–C magnification = 2×, Figs. 2D, E magnification = 4×, Figs. 2G–I magnification = 10×. Fig. 2F magnification = 40×.

**Figure 3:**
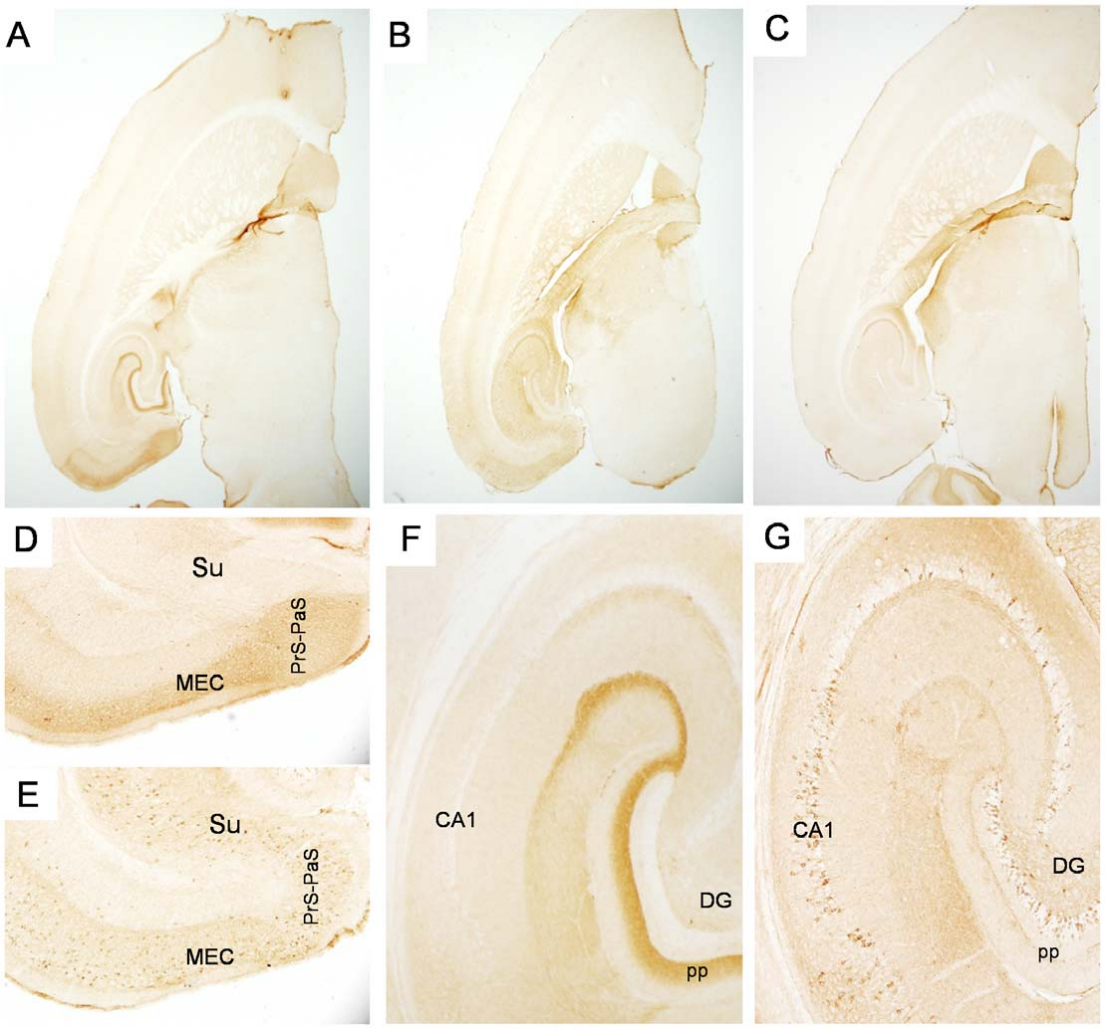
Progressive spread of tauopathy in NT mice identified by antibody CP27. Fig. 3 shows mice immunolabeled with CP27 (total human tau) at low power in a young NT mouse (Fig. 3A), an old NT mouse (Fig. 3B) or a control mouse (Fig. 3C). Higher power images from the young NT mouse (Figs. 3D, F), old NT mouse (Figs. 3E, G) with antibody CP27. CP27 is one of several antibodies that show non-specific staining of mossy fibers in the control mouse. Figs. 3D and E show the EC and subiculum whereas Figs. 3F and G show the CA and DG regions of the hippocampus. Figs. 3A–C magnification = 2×, Figs. 3D–G magnification = 10×.

A detailed histopathological analysis was performed on relatively young (10–11 mon) and aged (∼22 mon) neuropsin-tTA-tau (NT) transgenic mice using three different antibodies, MC1, CP27 and AT8. The MC1 monoclonal antibody detects human tau in an abnormal conformation [6] that is associated with early stage NFT–tau in human AD patients [7]. In young NT mice (Figs. 2A, D, G), abnormal human tau recognized by MC1 was most abundant in the medial EC (MEC). Relatively dense staining was also seen in the lateral EC (LEC) and the para-(PaS) subiculum while the presubiculum (PrS) was less intensely stained. Dense staining was seen in superficial layers II and III of the EC, whereas deeper layers showed considerably less staining. Human tau was present in some cell bodies, but mostly in neurites within the superficial layers of the MEC and LEC (Fig. 2G). Dense tau staining was seen in the middle third of the molecular layer of the DG and CA3 (Fig. 2D) but not the outer layer indicating tau in axon terminals of the perforant pathway (pp) that originate from layer II of the MEC [8]. In the CA1 and subiculum, the outer molecular layer was labeled extensively (Fig. 2D), indicating tau in perforant path terminals from layer III cells in both LEC and MEC [8]. Mice expressing only the uninduced tau transgene (control) showed negligible (Fig. 2C), or very limited immunoreactivity with the antibodies used, and it was usually restricted to the mossy fibers (for example, antibody CP27, Fig. 3C). Some non-specific staining in the fornix was seen in all mice, with all antibodies. By 22 months of age, the distribution of human tau in old NT mice had changed dramatically to resemble that seen in more affected AD brain tissue (Braak stages II–III). Intense MC1 immunoreactivity was readily detected not only in neurons in the superficial layers of the EC and throughout the subiculum (Figs. 2B, E, H), but in pyramidal neurons in the hippocampus, especially in CA1, and also in dentate gyrus granule cells (DG GCs) (Fig. 2E). Somatodendritic staining with MC1 was intense for cells in the MEC (Fig. 2F). Scattered MC1 positive neuronal cell bodies could also be seen in the perirhinal and the parietal cortices (Fig. 2I), and more extensively in the deeper layers of the EC.

**Figure 4:**
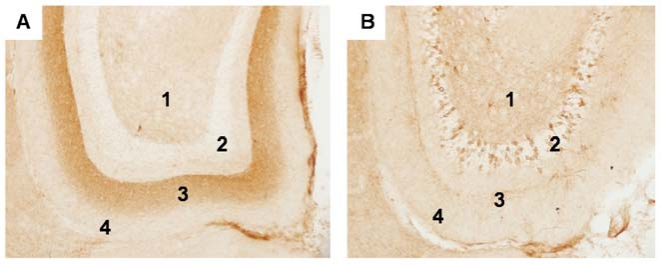
Progressive spread of tauopathy to monosynaptically connected regions of the hippocampus. Young NT mice (Fig. 4A) show accumulation of human tau immunolabeled with CP27 predominately in the endzones of the perforant pathway that terminate in the middle third of the molecular layer of the DG (area 3). Terminals from neurons in the LEC terminating in the outer third of the molecular layer are shown in area 4. Human tau was also seen in cells in the hilus (area 1). Granule cell layers of the DG (area 2) did not accumulate human tau at this age. Old NT mice (Fig. 4B) show accumulation of human tau in cell bodies in the granule cells of the DG (area 2). Increased accumulation of human tau is seen in layers 1, 2 and 4 but the perforant pathway endzone in layer 3 was significantly depleted of tau. Magnification = 20×.

The pattern of staining was reproduced in young and old NT mice using a human specific tau antibody (CP27) that recognizes all human tau, regardless of phosphorylation or conformation status (Fig. 3). Subtle differences in the relative intensity of staining in different areas were observed for different antibodies, especially in the DG GC layer where CP27 staining was more intense and extensive than MC1 (Figs. 3G and 4B vs. Fig. 2E). This could either indicate differential sensitivity of the antibodies, differential synthesis or clearance of tau forms recognized by the two antibodies, or retarded development of the conformational change in tau recognized by MC1.

To assess whether tauopathy could spread across a synapse, we examined cells in the DG that are monosynaptically connected with cells in the EC (Fig. 4). Young NT mice (Fig. 4A) showed robust accumulation of CP27 immunoreactive human tau in the endzones of the perforant pathway that originate from neurons in the MEC and terminate in the middle third of the molecular layer of the DG (area 3). Low, but detectable levels of immunoreactivity were seen in the outer third of the molecular layer (area 4) which represents terminals from neurons originating in the LEC. Some human tau was seen in cells in the hilus (area 1), most likely in mossy cells. Notably, human tau did not accumulate in DG GCs (area 2) in young NT mice. Old NT mice however showed a very different distribution of human tau (Fig. 4B). Robust accumulation of human tau was now seen in DG GCs (area 2) and increased accumulation of human tau was seen in layers 1, 2 and 4. The appearance of tau in DGGCs strongly supports the idea that tauopathy initiated in the EC can spread between cells that are connected, but physically separated by a synapse. Interestingly, the perforant pathway endzone in layer 3 was significantly depleted of tau which coincided with accumulation in originating cell bodies in the MEC (Fig. 2H, 2F). This apparent relocalization of tau from axons to somatodendritic compartments is one of the earliest events in the pathological cascade of early Alzheimer's disease [1].

**Figure 5:**
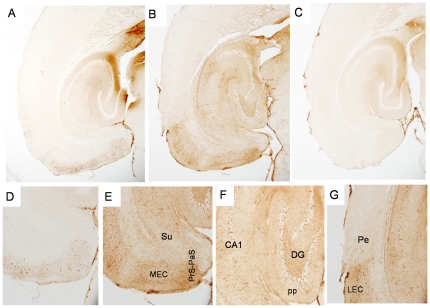
Progressive spread of tauopathy in NT mice identified by antibody AT8. Fig. 5 shows mouse brain tissue immunolabeled with AT8 (phospho-tau S202/205) at low power in a young NT mouse (Fig. 5A), an old NT mouse (Fig. 5B) or a control mouse (Fig. 5C). Higher power images from the young NT mouse (Fig. 5D), or old NT mouse (Figs. 5E–G) with antibody AT8. Figs. 5D and E show the EC and subiculum whereas 5F shows the CA and DG regions of the hippocampus. Fig. 5G shows the boundary between the LEC and the perirhinal cortex in the old NT mouse. Note the scarcity of neurite staining in the perirhinal cortex compared to the LEC. Figs. 5A–C magnification = 4×, Figs. 5D–G magnification = 10×.

Tauopathy in AD is usually staged using the antibody AT8 [9]. This antibody recognizes phosphorylated epitopes S202/205 (in both mouse and human tau) that are abundant in tau from AD brain, but not normal brain [10]. In young NT mice, (Figs. 5A, D) AT8 immunoreactive tau was mainly concentrated in the EC with no staining visible in the hippocampal subfields. Cell body staining was predominant with relatively less staining seen in neurites. In old NT mice (Figs. 5B, E, F, G), there was considerably more neurite staining throughout the EC (Fig. 5B, E), and in all fields of the hippocampus (Fig. 5F), with cell body immunoreactivity being seen in scattered neurons that were most prominent in pyramidal cells in the CA1 and in DG GCs. As for MC1, in the old mice, additional cell body staining was apparent in the deeper layers of the EC, and in cells in the perirhinal and parietal cortices (Fig. 5G). The control mouse (Fig. 5C) was essentially negative for this antibody. Overall, the pattern of staining, including extensive staining of cell bodies and neurites throughout the EC and hippocampus was reminiscent of that described for early Braak stages of AD [9].

**Figure 6:**
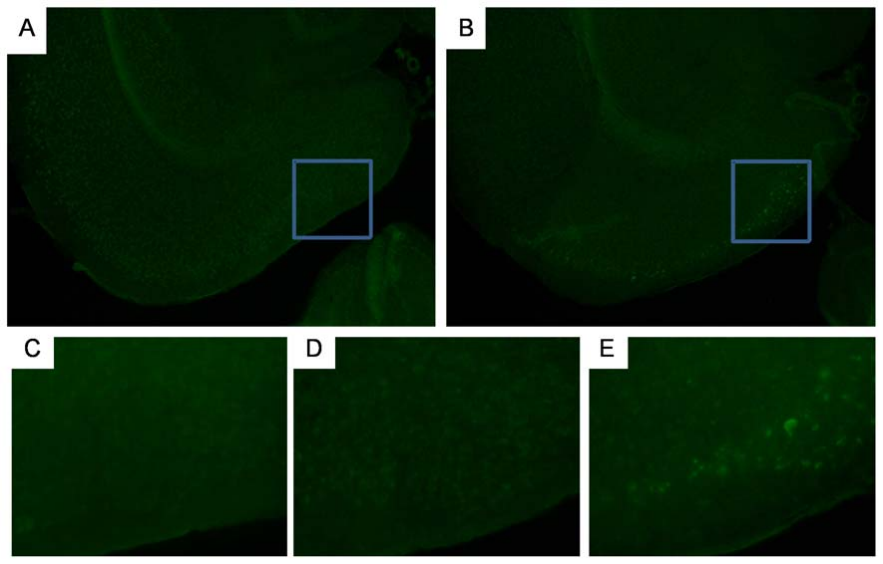
Mature, filamentous neurofibrillary tangle formation in the EC of old NT mice. Fig. 6A shows lack of thioS staining of neurons in the EC of an old control mouse. The boxed area is shown in the high power image in Fig. 6C. Fig. 6B shows thioS positive neurons in the EC of an old NT mouse. The boxed area is shown in the high power image in Fig. 6E. Fig. 6D shows lack of thioS staining in the same region of the young NT mouse. Figs. 6A and B magnification = 10×, Figs. 6C–E magnification = 20×.

Although the exact type of tau associated with functional impairment and degeneration is not known [11], the accumulation of insoluble, conformationally abnormal, hyperphosphorylated tau into mature neurofibrillary tangles in the somatodendritic cell compartment is generally associated with more severe pathology, degeneration and cell death. To test whether mature tangles had formed in the NT mice, we examined tissue sections stained with thioflavin S (thioS), a dye that binds to proteins in a β- sheet conformation, indicative of tau in mature tangles (Fig. 6). Special care was taken to mask lipofuscin fluorescence which is significant in old mice. A small number of neurons restricted to the MEC were positive for thioS in old NT mice (Figs. 6B, E). Young NT (Fig. 6D) and old control mice (Figs. 6A, C) were essentially negative. Not all of the tau immunoreactive neurons in the MEC of old NT mice were thioS positive, and cells in the LEC, CA1 and DG GC layer were thioS negative, as were neurites and axonal terminals in the perforant pathway. As cells with the highest level of human tau occur in the MEC, the lack of staining in other areas is most likely explained by the lower tau levels rather than by regional sensitivity to tangle formation, but the latter interpretation cannot be ruled out in these studies.

**Figure 7:**
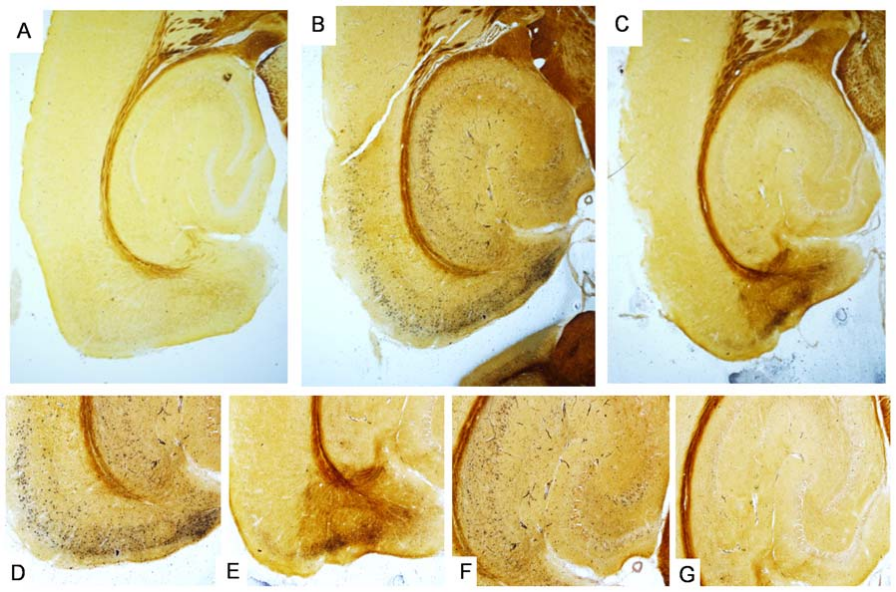
Silver staining in NT mice. Fig. 7A shows a lower power image of argyrophilic material in the hippocampus and cortex of a young NT mouse (Fig. 7A), an old NT mouse (Fig. 7B) and a control mouse (age matched to the old NT mouse) (Fig. 7C). Higher power images from the EC and subiculum of the old NT mouse (Fig. 7D) or control mouse (Fig. 7E). Figs. 7F and G show high power images of argyrophilic neurons in the hippocampus of the old NT mouse (Fig. 7F) or control mouse (Fig. 7G). Note the relatively abundant, dense staining in EC, subiculum and CA regions compared to the faint staining in the DG, which mirrors the staining pattern with MC1. Figs. A, B and C magnification = 4×, Figs. 7D–G magnification = 10×.

Altered conformation of proteins can also be visualized by silver staining using one of several methods [12]. Argyophilic plaques, tangles and neurites are abundant in the human AD brain. Abundant, argyrophilic cell body and neurite staining was also seen in the old (Figs. 7B, D, F), but not the young NT mice (Fig. 7A), and it was related to tauopathy development rather than aging as parallel-processed, old littermate control mice were negative (Figs. 7C, E, G). The distribution of histopathology in the old NT mouse was extensive, with robust staining being seen in cells in the EC, as well as in the subiculum (Fig. 7D). Staining was also extensive in the CA1, but to a lesser extent in the DG GC layer (Fig. 7F). In general, the distribution of silver-staining matched that seen with the MC1 antibody more closely than that seen with the CP27 or AT8 antibodies, suggesting that it is the conformational change in tau recognized by MC1 that is recognized by the silver stain. Interestingly, MC1 immunoreactivity was robust in neurites in the young NT mice but these mice were negative for silver staining. Therefore the silver stain recognizes a more advanced conformational abnormality that lies between pre-tangle MC1 immunoreactivity seen in the young mice, and the overt conformational change recognized by thioS, which in the old mice, is restricted to cells in the MEC.

### Trans-synaptic spread of pathology identified, but mechanism unknown

**Figure 8:**
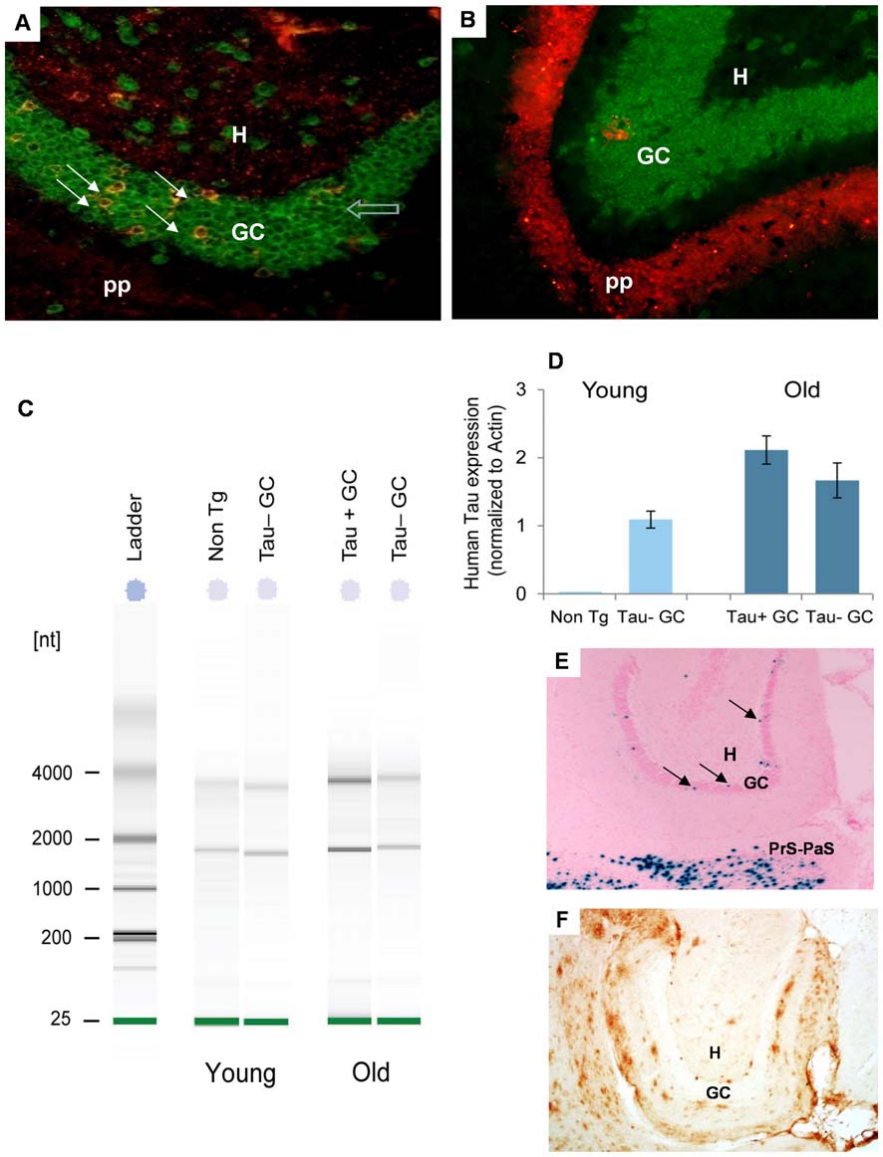
LCM-isolation of cell populations and assessment of mRNA levels by qRT-PCR. Fig. 8A shows high power images of frozen sections from an old NT mouse double-immunolabeled with antibodies recognizing the neuronal marker NeuN (green) and human tau CP27 (red), before LCM capture of dentate gyrus granule cells (GCs). Co-localization between CP27 and the neuronal marker NeuN indicates that expression of human tau was neuronal in origin and glial expression was negligible, or absent. Human tau positive neurons (Tau+ GC, yellow cells, solid white arrow) and tau negative neurons (Tau− GC, green cells, open blue arrow) were isolated. Fig. 8B shows the GC layer from a young mouse. Granule cell neurons in the DG were not immunoreactive for human tau at this age therefore only green neurons (Tau− GCs) in this region were isolated by LCM. Fig. 8C shows a gel image from the Agilent Bioanalyzer of RNA extracted from LCM isolated cells. Fig. 8D shows human tau expression in LCM isolated non Tg and Tau− GC of a young animal and in Tau+ GC and Tau− GC neurons from an old NT mice. The relative expression changes (2^−ΔΔCT^) between the cell types were calculated after normalization to β-actin expression. Error bars represent the SD for triplicates of each RT-cDNA sample used for qPCR. One old and one young mouse are represented in Fig. 8D; analyses were performed on cells from three individual old mice, and two young mice. Fig. 8E shows the DG of the neuropsin-tTA-lacZ reporter gene mouse (3 months of age) with GCs expressing the responder indicated (arrows). Fig. 8F shows the DG of a neuropsin-tTA-APP mouse with the DG GCs indicated. Mice were 22 months of age; two mice were examined. Despite the presence of extensive plaque pathology in adjacent layers, no APP/Aβ staining was seen in the DG GCs from this mouse line. Figs. 8A, B magnification = 20×. pp = perforant pathway endzone, H = hilus, GC = dentate gyrus granule cells.

One of the most intriguing observations from our studies is the appearance of tauopathy in cells outside of the entorhinal cortex. As shown in Fig. 4B, granule cells in the DG of old NT mice accumulate human tau protein, but it is unknown whether the human tau protein accumulated in the DG GCs derives solely from uptake or transfer of human tau from neurons in the EC, or if the human tau protein could be generated from endogenously produced human tau mRNA in the DG GCs resulting from “leaky” expression of the transgene. To test whether endogenously produced human tau in the DG GCs could be contributing to the tauopathy seen there, we collected approximately 1000 individual neurons by laser-capture microdissection (LCM) from the DG GC layer from old (Fig. 8A) and young (Fig. 8B) mice and assessed whether human tau mRNA was expressed in them. For this experiment, young mice at ∼4 months of age were sampled to reduce the likelihood that incipient pathology had developed. Old NT, young NT and non transgenic mouse tissue sections were double immunolabeled with both CP27 and NeuN to ensure that neurons were isolated. Fig. 8C shows total RNA extracted from LCM isolated cell populations from one mouse from each type. A gel image from the 2100 Bioanalyzer, which employs capillary gel electrophoretic methodology to measure RNA integrity (RIN) and abundance, demonstrated the quality of sample from the LCM isolated cells in young mice - Non Tg (RIN 6.3; 418 pg/µl; total yield 4.18 ng), tau protein negative (Tau−) GCs (RIN 8.1; 443 pg/µl; total yield 4.43 ng), and old mice - tau protein positive (Tau+) GCs (RIN 8.6; 907 pg/µl; total yield 9.07 ng) and Tau− GCs (RIN 7.8; 351 pg/µl; total yield 3.51 ng). The level of human tau mRNA was then measured by quantitative (q) RT-PCR. To confirm the specificity of our human tau primers, LCM was used to isolate DG GCs from a non Tg young mouse that had been processed in the same way as the young NT mouse. QRT-PCR identified no amplification from the non Tg sample (C_T_>40) demonstrating that the primers were completely human specific (Fig. 8D). To assess whether amplification could result from residual DNA contamination even after DNAase treatment, RNA from DG GCs was used for qPCR with, or without the reverse-transcription step required to synthesize cDNA. Human tau amplicons in the sample that had not been reverse-transcribed were found to be at least twenty five fold (25X) lower than in the transcribed samples, suggesting that genomic DNA transgene priming resulted in only very low levels of PCR amplification compared to mRNA priming. Fig. 8D shows human tau expression in the human tau protein negative DG GCs (Tau− GCs) from a young NT mouse (left side of panel), and in human tau protein positive (Tau+ GCs) and negative (Tau− GCs) DG GCs from an old NT mice (right side of panel). Surprisingly, in the young NT mouse where all of the DG GCs were tau protein negative, the Tau− GCs had low but detectable levels of human tau mRNA after normalization to β-actin. In old mice, human tau mRNA was again detected in DG GCs from the NT mouse, both in cells that were positive or negative for tau protein. Relative expression changes (2^−ΔΔCT^) between the two populations of DG GCs in the old NT mouse showed a 27% increase in expression levels between Tau− and Tau+ DG GCs. A second, old NT mouse also had quantifiable levels of human tau mRNA in all three cell populations, with mRNA levels in Tau+ GCs>Tau− GCs (data not shown). The reason for this apparent increase is unknown but if it is significant in a larger sample group, it could reflect a feedback mechanism whereby tau transcription is upregulated if tau is losing its ability to perform its' normal function as it accumulates in the cytosol in the Tau+ DG GCs. The apparent increase in tau levels in old compared to young samples could reflect an age-dependent increase in transcription, or it could simply represent inter-animal variability.

Despite the absence of detectable human tau protein immunoreactivity in the tau negative DG GCs from young or old NT mice; human tau mRNA was identified in cells from three separate experiments suggesting that transgene expression was slightly leaky. To further examine whether ectopic expression in the DG GCs could explain the accumulation of protein at old age, we examined mice from two other crosses to the neuropsin tTA activator mouse. The first cross was to a reporter gene expressing LacZ with a nuclear localization signal to restrict the cellular distribution of the reporter to the nucleus of the cell in which it was produced [13]. As shown in Fig. 8E, positive staining for LacZ was restricted to very few cells in the DG GC layer. The second cross was to a mutant APP responder line. When crossed to the neuropsin-tTA activator, this mouse expresses APP predominantly in the EC as expected (data not shown), but due to secretion of Aβ, plaques accumulate in regions outside of the EC, including layers of the DG. Of note, the cells of the DG GC layer did not accumulate APP/Aβ supporting our observations that ectopic expression of responder genes in this cell layer is negligible. Therefore, despite our findings of some human tau mRNA in DG GCs, ectopic expression in these cells is very limited and unlikely to account for the extensive immunolabeling with human tau specific antibodies seen in the old mice. As we pooled many DG GCs and analysis of mRNA levels by qPCR is extremely sensitive, the actual number of cells contributing the human tau mRNA could be a very low percentage of the total that were human tau protein positive. How human tau protein accumulating in DG GCs that were likely to be human tau mRNA negative was derived is unknown, but it is possible that tau was released from cells originating in the EC, and internalized by DG GCs synapsing on to them. In support of this mechanism, a recent study has shown that tau can be released from cells via exosomes and tau positive exosomes have been identified in human CSF from AD patients [14].

In general, our NT mouse model replicates the spatial and temporal aspects of the earliest stages (I–III) of Braak staging of tauopathy in Alzheimer's disease. We have demonstrated that tau pathology initiating in the EC can spread to other synaptically connected brain areas as the mice age, supporting the idea that AD progresses via an anatomical cascade as opposed to individual events occurring in differentially vulnerable regions. Thus, our NT transgenic mouse provides a model in which the spatial and temporal propagation of the disease can be predicted, and correlative functional outcomes can now be tested. Given that the earliest Braak stages are not associated with cognitive decline, identifying an EC based “biomarker” for pathology or dysfunction and developing therapeutic strategies to prevent propagation are likely to be both possible, and beneficial.

## Materials and Methods

### Ethics statement

Animals were used in full compliance with the National Institutes of Health/Institutional Animal Care and Use Committee guidelines. The protocol was approved by the Committee on the Ethics of Animal Experiments of Columbia University under protocol # AC-AAAB7457.

### Transgenic mice

To achieve differential expression in the entorhinal cortex we have used an inducible mouse line (neuropsin-tTA; the “activator”) in which the tTA activator is driven by the promoter for the neuropsin gene [15]. This results in robust expression of tTA in the EC, primarily in superficial layers of the MEC, and to a lesser extent in the LEC and pre- and para-subiculum. Neuropsin-tTA-Tau (NT) mice were generated by crossing the neuropsin-tTA activator line with a tetracycline inducible, tau Tg mouse, line rTg4510 (the “responder”). This line consists of a tetracycline-operon–responsive element (TRE) placed upstream of a cDNA encoding human four-repeat tau with the P301L mutation (4R0N tauP301L) [11]. Immunohistochemical analysis of neuropathology has been performed on n = 4−5 mice from each age group with consistent results. Two other responder lines were crossed to the neuropsin-tTA line to test for ectopic expression of the transactivator protein in the DG. The second responder line consists of the TRE placed upstream of a cDNA encoding a nuclear localized fusion of lacZ and green fluorescent protein [13]. Data from mice at 3 months of age was supplied by Dr. Joanna Jankowsky. The third responder line expresses mutant APP (line B6.Cg-Tg (tetO-APPSwInd) 885 Dbo/J). This APP line was obtained from Jackson laboratories and crossed to the neuropsin-tTA line. Data shown is from mice at 22 months of age. All figures show one mouse from each group.

### Immunohistochemistry

Mouse brains were harvested after transcardial perfusion and drop-fixed in 4% paraformaldehyde overnight, followed by cryoprotection treatment in 30% sucrose in PBS for 16 hours. Free-floating brain sections (30 µm) from brains sectioned in the horizontal plane were used for immunohistochemistry using SuperPicTure™ polymer detection kit (Zymed, San Francisco, CA). Sections were washed with PBS for 10 minutes, and then treated with 3% H_2_O_2_ in PBS for 10 minutes. The sections were then transferred to a microfuge tube that contained 1 ml of primary antibody diluted in PBS containing 0.3% Triton and 5% normal serum, and incubated at 4°C overnight on a rotator. After three washes with PBS-T (0.1% Triton-X 100), the sections were incubated for 10 minutes with HRP polymer conjugate. Following three washes with PBS-T, immunoreactive material was visualized using DAB as chromagen. The stained sections were mounted on slides and inspected by light microscopy. Three monoclonal antibodies were used for detection of tau. MC1 (gift of Dr. Peter Davies) detects an abnormal conformational epitope of tau that is associated with NFT formation [7]. CP27 is specific for human tau (conformation and phosphorylation status independent, gift of Dr. Peter Davies). AT8 recognizes tau phosphorylated at S202/205, in mouse and human tau [10]. Staining for APP/Aβ shown in Fig. 8F was performed on horizontal sections from 22 month old neuropsin-tTA-APP mice, as described for tau immunohistochemistry, using the 6E10 antibody (Covance Inc.) which recognizes human APP/Aβ.

### LacZ staining

Mouse brains were harvested at 3 mo of age after transcardial perfusion and drop-fixed in 4% paraformaldehyde for 2 hours, followed by cryoprotection in 30% sucrose/1x PBS for 24 hours. Horizontal sections (35 µm) were mounted and allowed to air dry for 30 minutes prior to brief incubation in PBS+2 mM MgCl_2_. Slides were then pretreated in 0.1 M NaPO_4_, 2 mM MgCl_2_, 0.2% NP40, and 0.1% sodium deoxycholate, before being developed in a 0.6% X-Gal (5 Prime™)/10 mM potassium ferri/ferrous cyanide solution in the dark for 1 hr at 37 C [16]. Development was stopped with 1× HEPES buffered saline, 0.1% Triton X, 1 mM EDTA, followed by a 1 hr post fixation with 4% paraformaldehyde. Sections were counterstained with Nuclear Fast Red, dehydrated through alcohol, and mounted in a xylene-based medium.

### Thioflavin-S staining

To reduce the lipofuscin fluorescence background staining in aged mice, a modified thioflavin-S staining protocol [17] was used as follows. Free floating brain sections were incubated in 0.3% KMnO_4_ for 3–5 minutes, washed with water, then treated with a solution of 1% K_2_ S_2_O_5_ and 1% oxalic acid until the brown color was removed from the tissue (20–40 secs). Sections were then stained with 0.05% thioflavin-S in 50% ethanol in the dark for 8 minutes, followed by differentiation in two changes of 80% ethanol for 10 secs each and three washes in large volumes of distilled water. The sections were mounted and coverslipped with VectaShield mounting medium (Vector Lab), and observed with a fluorescence microscope.

### Bielschowsky silver staining

Brain sections (30 µm) prepared as for IHC were mounted on slides and silver stained according to Bielschowsky's method as described [12].

### Immuno Laser-Capture Microdissection (LCM)

Mouse brains were rapidly removed after cervical dislocation, and were progressively frozen by slow immersion (∼60 sec) in dry ice chilled isopentane. The frozen brains were embedded in O.C.T. and sectioned horizontally at 10 µm. The sections were mounted on pre-treated membrane slides (according to manufacturer's manual, Carl-Zeiss MicroImaging, LLC) and stored at −80°C before use. CP27 (1∶25) antibody was used to label human tau positive neurons and Alexa Fluor 568 was used as a secondary antibody (1∶25). For double-immunolabeling, sections were incubated with CP27 as before, and with NeuN polyclonal antibody (1∶25, Millipore) with Alexa Fluor 488 used as secondary antibody (1∶25). Antibodies were diluted in PBS containing 200 U/ml of RNAse inhibitor. All staining procedures were performed on ice, using RNase-free conditions. The brain sections were fixed in acetone for 3 minutes, followed by a PBS wash for 30 secs, then incubated with CP27 antibody for 10 minutes. After rinsing the slides in PBS (10 dips), secondary antibody was applied for 2 minutes. Slides were briefly rinsed with PBS and H_2_O, and dehydrated through 70%, 95%, and 100% Ethanol (10 dips each). The slides were kept at room temperature and used for LCM within 90 minutes. Human tau positive neurons were identified using fluorescence microscopy, laser captured with a PALM MicroLaser Systems (Carl-Zeiss MicroImaging, LLC) and collected using AdhesiveCaps. One thousand cells were captured from each region and used for quantitative RT-PCR analysis.

### Quantitative RT-PCR (qPCR)

RNA was extracted from each LCM cell population using the ArcturusPicoPure RNA Kit (Applied Biosystems, Foster City, CA), also with a DNase I treatment step. RNA extracted from LCM isolated cells was analyzed using a 2100 Bioanalyzer (Agilent, Technologies, Santa Clara, CA). 2.5 ng of RNA was employed for first strand cDNA synthesis, in a 20 µl reaction volume using a 1∶1 ratio of random hexamer and oligo-dT primers and Superscript® III RT enzyme (Life Technologies Inc., Carlsbad, CA). cDNA products were diluted with 70 µl RNAse free water, prior to quantitative PCR of the human tau transgene and the housekeeping gene β-actin. PCRs were performed by monitoring in real time the increase in fluorescence of the SYBR Green dye, using a Bio-Rad iQ5 detector system (Bio-Rad, Hercules, CA). Primers specific to the human transgene (5′-CCCAATCACTGCCTATACCC-3′ and 5′-CCACGAGAATGCGAAGGA-3′ as published [11]), or β-actin (5′-GCTCTTTTCCAGCCTTCCTT-3′ and 5′-AGTACTTGCGCTCAGGAGGA-3′) were employed in triplicate in 20 µl PCR reactions using 250 nM each primer pair, 8 µl of diluted cDNA and 10 µl of iQ SYBR Green Supermix, using amplification conditions as follows: 95°C for 3 minutes, followed by 40 cycles of denaturation at 95°C (10 secs), priming at 60°C (30 secs), and extension and fluorescence capture at 72°C (30 secs). A melt curve was also employed to test the specificity of each primer pair.

A standard curve was generated for each gene, using dilutions of cDNA synthesized from a control RNA sample extracted from mouse cortex: 100 ng cDNA, 1 ng cDNA and 0.1 ng of cDNA input. Relative expression changes were calculated using the 2^−ΔΔCT^ method, where the C_T_ is defined as the fractional cycle number in which the template amplification reaches a fixed threshold [18]. Data were analyzed in GraphPad Prism v5.
